# Correlation between polio immunization coverage and overall morbidity and mortality for COVID-19: an epidemiological study

**DOI:** 10.1007/s11356-021-12861-6

**Published:** 2021-03-02

**Authors:** Marwa Adel Afify, Rakan M. Alqahtani, Mohammed Abdulrahman Mohammed Alzamil, Faten Abdulrahman Khorshid, Sumayyah Mohammad Almarshedy, Sana Ghazi Alattas, Talal Nabeel Alrawaf, May Bin-Jumah, Mohamed M. Abdel-Daim, Mohammad Almohideb

**Affiliations:** 1 Potion CRO, Integrative Medicine Company, Al Malqa, Riyadh, 13524 Saudi Arabia; 2 Department of Critical Care Medicine, College of Medicine, King Saud University, Riyadh, Saudi Arabia; 3 Prince Sultan Medical Military City (PSMMC), Riyadh, Saudi Arabia; 4 Department of Biological Sciences, Faculty of Science, King Abdulaziz University, Jeddah, 21589 Saudi Arabia; 5 Division of Adult Neurology, Department of Internal Medicine, College of medicine, University of Hail, Hail, Saudi Arabia; 6 Biological Sciences Department, King Abdulaziz University, Jeddah, Saudi Arabia; 7 Al Yamamah Hospital, Riyadh, Saudi Arabia; 8 Biology Department, College of Science, Princess Nourah Bint Abdulrahman University, Riyadh, Saudi Arabia; 9 Department of Zoology, Science College, King Saud University, Riyadh, 11451 Saudi Arabia; 10 Pharmacology Department, Faculty of Veterinary Medicine, Suez Canal University, Ismailia, 41522 Egypt; 11 College of Medicine, King Saud bin Abdulaziz University for Health Sciences, Riyadh, Saudi Arabia

**Keywords:** Polio, Immunization, COVID-19, Correlation

## Abstract

We conducted the current analysis to determine the potential role of polio vaccination in the context of the spread of COVID-19. Data were extracted from the World Health Organization’s (WHO) Global Health Observatory data repository regarding the polio immunization coverage estimates and correlated to the overall morbidity and mortality for COVID-19 among different countries. Data were analyzed using R software version 4.0.2. Mean and standard deviation were used to represent continuous variables while we used frequencies and percentages to represent categorical variables. The Kruskal-Wallis *H* test was used for continuous variables since they were not normally distributed. Moreover, the Spearman rank correlation coefficient (rho) was used to determine the relationship between different variables. There was a significantly positive correlation between the vaccine coverage (%) and both of total cases per one million populations (rho = 0.37; *p*-value < 0.001) and deaths per one million populations (rho = 0.30; *p*-value < 0.001). Moreover, there was a significant correlation between different income groups and each of vaccine coverage (%) (rho = 0.71; *p*-value < 0.001), total cases per one million populations (rho = 0.50; *p*-value < 0.001), and deaths per one million populations (rho = 0.39; *p*-value < 0.001). All claims regarding the possible protective effect of Polio vaccination do not have any support when analyzing the related data. Polio vaccination efforts should be limited to eradicate the disease from endemic countries; however, there is no evidence to support the immunization with live-attenuated vaccines for the protection against COVID-19.

**Supplementary Information:**

The online version contains supplementary material available at 10.1007/s11356-021-12861-6.

## Introduction

As of December 2019, a significant number of patients with “unknown viral pneumonia” connected to a local Seafood Wholesale Market were identified in Wuhan City, China (Huang et al. 2020). A novel coronavirus strain (SARS-CoV-2) was recognized as the cause of the 2019 coronavirus disease (COVID-19). SARS-CoV-2 has exhibited an unprecedented spreading potential, affecting more than 210 countries all over the globe. However, there are dramatic differences in patterns of disease spread and how it behaves in various countries. For example, as of 17 March 2020, the UK and Italy have strikingly high mortality rates of 13.41% (total cases = 108.692) and 13.19% (total cases = 172.434), respectively, despite the deployment of extensive restrictions on social interactions in these countries. On the other hand, the USA, which accounts for 29.5% of the total confirmed cases of COVID-19 globally, has a low mortality rate of 5.27%, which is the case in many other countries (Glass et al. 2004). These differences have been hypothesized to be attributable to the variations in cultural norms and healthcare infrastructure. Herein, we propose an alternative theory, suggesting that the differences in the number of COVID-19 cases and the associated mortality rates might be explained, in part, by the variations in the coverage rates of universal polio vaccination policy in different countries all over the world.

Oral poliovirus vaccine (OPV) and inactivated poliovirus vaccine (IPV) have been used to eradicate poliovirus. In addition to the protective properties of live-attenuated vaccines against specific microorganisms, various vaccines have been shown to induce non-specific immune effects. Recent epidemiological data suggest that the use of live-attenuated vaccines (for example, Bacillus Calmette-Guérin (BCG), measles vaccine, and OPV) may result in the induction of non-specific effects in the immune system (Blok et al. 2015; De Bree et al. 2018; Higgins et al. 2014; Jensen et al. 2016) and may protect against other types of viruses (Aaby and Benn 2017; Higgins et al. 2016; Upfill-Brown et al. 2017). These non-specific effects are mediated via various immunological mechanisms, including the induction of the innate immune system (trained immunity) and heterologous lymphocyte effects. In turn, trained immunity promotes the long-term upregulation of innate immune cells via epigenetic and metabolic programming (De Bree et al. 2018). Even though the exact mechanisms behind this phenomenon—trained immunity and non-specific immune effects—are not yet clearly understood, we conducted the current analysis to determine the potential role of polio vaccination in the context of the spread of COVID-19 until specific vaccines and antiviral therapy are designed and approved.

## Methods

### Data collection

Data were extracted from the World Health Organization’s (WHO) Global Health Observatory data repository regarding the polio immunization coverage estimates (González et al. 2003). The coverage was estimated as “the percentage of children ages 12–23 months who received three doses of the polio vaccine before the survey” (Li et al. 2016b). Data of COVID-19 total cases per one million populations and deaths per one million populations, for all possible countries, were obtained from the Internet continuously updated repository “Worldometers” on 28 October 2020 (Karimi et al. 2020). Finally, the data were then merged according to the country to restore only countries having reported immunization coverage.

### Statistical analysis

Mean and standard deviation (SD) were used to represent continuous variables while we used frequencies and percentages to represent categorical variables. The skewness and Kurtosis tests were used for testing the normal distribution of continuous variables. The Kruskal-Wallis *H* test was used for continuous variables since they were not normally distributed (Shapiro-Wilk *p*-value < 0.001 for all variables) (Chan and Walmsley 1997). Moreover, the Spearman rank correlation coefficient (rho) was used to determine the relationship between different variables (de Winter et al. 2016; Kumar et al. 2018). Data were analyzed using R software version 4.0.2 using the packages (Rcmdr) and (corrr). The statistical significance was considered when the *p*-value was < 0.05.

## Results

**Fig. 1:**
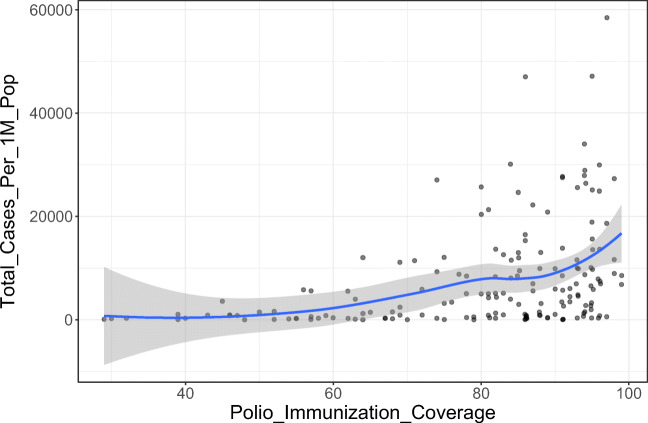
The correlation between polio immunization coverage (%) and new cases per one million populations

**Fig. 2:**
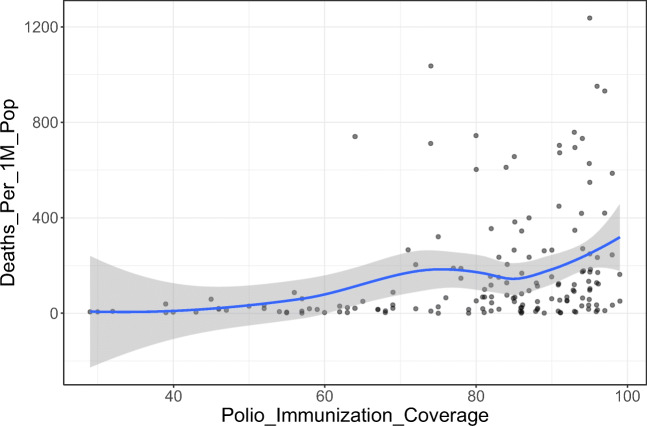
The correlation between polio immunization coverage (%) and deaths per one million populations

**Fig. 3:**
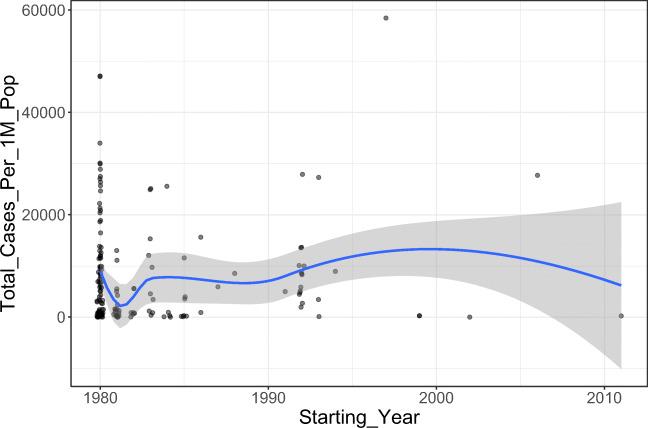
The correlation between starting year polio immunization policy and new cases per one million populations

**Fig. 4:**
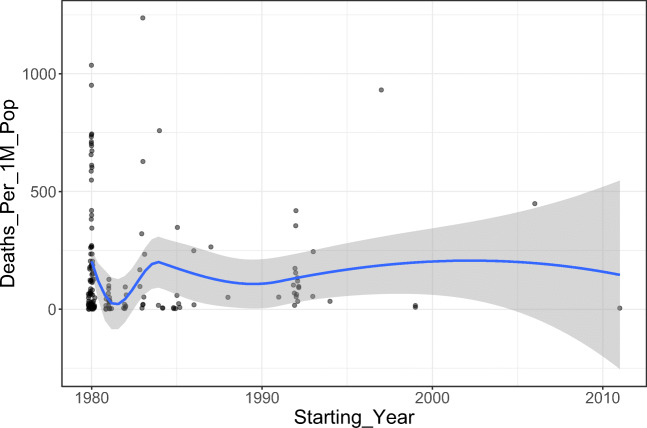
The correlation between starting year polio immunization policy and deaths per one million populations

Initially, we analyzed the correlation between polio vaccination coverage (%) and COVID-19 statistics in different countries (Tables 1 and 2). There was a significant positive correlation between the vaccine coverage (%) and both of total cases per one million populations (rho = 0.37; *p*-value < 0.001) and deaths per one million populations (rho = 0.30; *p*-value < 0.001) (Figs. 1 and 2). On further analysis of the effect of starting year of vaccination policy, there was a positive correlation with total cases per one million populations (rho = 0.07; *p*-value = 0.375) and a negative one with deaths per one million populations (rho = − 0.02; *p*-value = 0.788); however, these correlations were not statistically significant (Figs. 3 and 4).

**Table 1:**
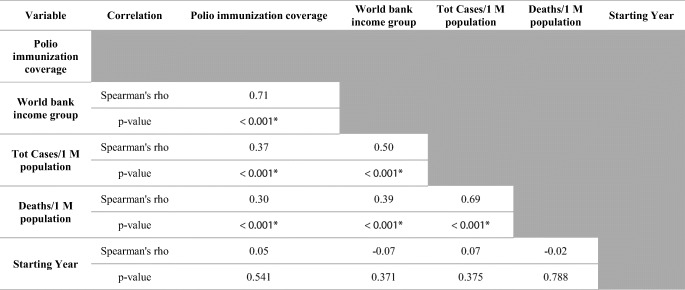
Correlation matrix between different variables

*Statistically significant *p*-value < 0.05

**Table 2:** Data stratified according to the World Bank income groups

World Bank income group	Polio immunization coverage (%)		Tot cases/1M pop		Deaths/1M pop	
	Mean	Standard deviation	Mean	Standard deviation	Mean	Standard deviation
High income	92.00	10.00	612.59	1023.01	11.80	11.76
Upper middle income	84.00	18.00	8569.45	8068.06	214.62	136.79
Lower middle income	71.00	26.00	3118.89	3739.36	84.64	136.79
Low income	60.00	27.00	13,871.58	13,037.06	256.36	291.62

**Fig. 5:**
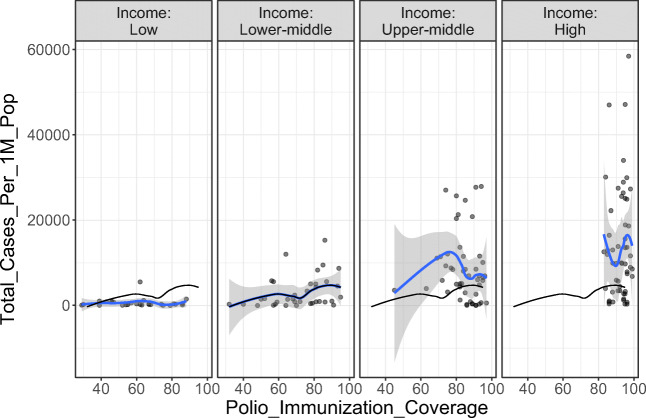
The correlation between polio immunization coverage (%) and new cases per one million populations (stratified by World Bank income group)

**Fig. 6:**
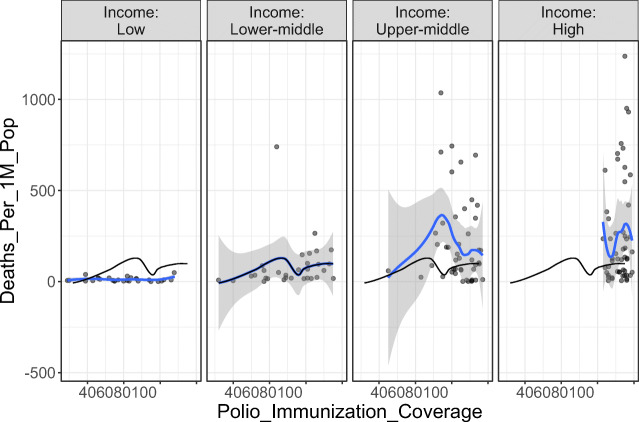
The correlation between polio immunization coverage (%) and deaths per one million populations (stratified by World Bank income group)

We furtherly did a stratification of data according to the World Bank income group (Table 2); in general, there was a significant correlation between different income groups and each of the vaccine coverage (%) (rho = 0.71; *p*-value < 0.001), total cases per one million populations (rho = 0.50; *p*-value < 0.001), and deaths per one million populations (rho = 0.39; *p*-value < 0.001) (Table 1). In the same context, there was a significant difference among different income groups in terms of polio immunization coverage (*X*^2^ = 90.96; *p*-value < 0.001), total cases per one million populations (*X*^2^ = 117.05; *p*-value < 0.001), and deaths per one million populations (*X*^2^ = 75.76; *p*-value < 0.001) (Figs. 5 and 6). Moreover, the pairwise comparisons between each pair of World Bank groups showed a significant difference among the aforementioned three aspects, except for “lower-middle” and “low” income groups which were comparable (*p*-value < 0.05) (Supplementary Tables).

## Discussion

Oral poliovirus vaccine (OPV) and inactivated poliovirus vaccine (IPV) are the two major types of vaccines that protect individuals against poliovirus. Polio is now recognized as the main vaccine-preventable disease after receiving the required attention and support for its complete eradication (Mshelia et al. 2020). Following the intensive efforts of the Global Polio Eradication Initiative (GPEI), which was launched in 1988 to reach the goal of complete polio eradication, the incidence of this disease was successfully reduced to 99% (Glass et al. 2004). Meanwhile, the current policy of GPEI in eradicating poliovirus is the Polio Endgame Strategy, which was set to take place between 2019 and 2023, with three major goals: polio eradication, integration, and certification and containment (World Health Organization 2019). However, soon enough after the emergence of a new coronavirus strain (COVID-19), all universal polio vaccination campaigns were suspended by the GPEI program until the 2nd half of 2020 in an attempt to reduce the risk of increasing transmission of COVID-19 through healthcare providers and laboratory personnel (Li et al. 2016a). Therefore, we conducted the current analysis to determine whether poliovirus vaccination with OPV or IPV has a protective role against COVID-19.

Live-attenuated RNA virus vaccines such as mumps, measles, polio, or rubella viruses are known to result in the induction of long-term immune response in terms of both cell-mediated and humoral immunity following one or two doses of these vaccines (Escriou et al. 2014). However, the question of whether the universal coverage of such vaccines might protect against or slow the spread of COVID-19 infection remains to be answered.

We conducted the current study as an attempt to identify the potential correlation between the coverage rates of universal poliovirus vaccines and the number of mortality cases as well as new cases of COVID-19 in different countries all over the globe to provide possible explanations for the variations in trends of disease spread all over the world. Overall, we noted that countries that had higher polio vaccine coverage rates were associated with higher COVID-19 disease burden in terms of mortality rates and the total number of COVID-19-confirmed cases per one million populations. Noteworthy, this finding was highly significant, indicating that polio vaccination does not protect against COVID-19 or slow its spread. Moreover, countries that implemented the universal polio vaccination policies earlier reported higher mortality rates and higher total numbers of COVID-19 cases (per one million population) compared to countries that implemented polio vaccination policies at later a date. This indicates that the earlier the universal polio vaccination policy was deployed, the greater the number of COVID-19 cases as well as the greater the number of mortality cases per one million populations.

Countries were divided into low, low-middle, upper-middle, and high-income countries based on the World Bank classification. We noted that low-income countries had the lowest mean poliovirus (3rd dose) vaccination coverage rates of 60% compared to the 92% mean coverage rate of high-income countries. Surprisingly, these low-income countries had much lower mortality cases compared to high-income countries (mean deaths per 1 million populations: 0.66 vs 89.16). In the same context, the total number of COVID-19 cases was dramatically lower in low-income countries compared to high-income ones (mean: 16.43 vs 1493.04 new cases per one million populations). These differences were noted to be highly significant, indicating that coverage of polio vaccination policies is not correlated with a protective effect against COVID-19 infection. However, it should be noted that a considerable proportion of low-income countries did not report the rates of mortality or report no deaths due to COVID-19, while nearly half low-income countries reported mortality rates < 1 per one million populations. Therefore, the aforementioned differences based on the income of countries could be attributed to the underreporting of these countries.

On 28 March 2020, Miller et al. (2020) conducted a similar analysis to determine if a potential correlation between universal Bacillus Calmette-Guérin (BCG) vaccination policies in different countries and the morbidity, as well as mortality associated with COVID-19 infection, exists and whether BCG vaccination is protective against or indicative of the disease burden in each country accordingly. In their analysis, the authors found that 55 middle-high and high-income countries with a current BCG vaccination policy had much fewer mortality cases per million populations when compared with five middle-high and high-income countries, where no universal BCG vaccination policies exist (mean mortality rate: 0.78 vs 16.39). In the same context, middle-high and high-income countries with no universal BCG vaccination policy have a fourfold increase in the number of COVID-19 cases per million populations. The authors also observed a significant correlation between the timing of universal BCG vaccination policy implementation and the number of mortality cases; they found that the earlier the vaccination policy was implemented, the fewer death cases were reported, indicating a possible protective effect of early BCG vaccination.

To date, there is no evidence that OPV protects against COVID-19 infection. Multiple experimental studies that have been conducted over the years have reported that OPV has non-specific effects on the immune system; all of which can potentially protect against COVID-19 in theory (Aaby and Benn 2017; Upfill-Brown et al. 2017). However, these effects have not been well-characterized, and their clinical relevance is not yet known. These reported effects may not be essentially limited to OPV, as various live vaccines have also been reported to exhibit non-specific protective effects in the immune system as well, such as the BCG vaccine against tuberculosis (Miller et al. 2020). The underlying mechanism of potential immune protection, through the use of these vaccines, needs to be elucidated further. The major advantage in this matter is that clinical studies can be initiated right away, as the vaccines in question are licensed and have excellent safety profiles. Polio vaccination efforts should be limited to eradicate the disease from endemic countries; however, there is no evidence to support the immunization with live-attenuated vaccines for the protection against COVID-19.

Our analysis does not account for a direct association between increased universal poliovirus vaccination coverage rates and increased rates of new COVID-19 cases and mortality rates per 1 million populations among analyzed countries. Many significant role-playing factors could have attributed to such results; however, we could not identify these factors due to the limited data available in this matter. We also could not analyze whether a certain type of polio vaccine (OPV or IPV) was the main contributor to our results because these data were not available. Notably, underreporting in low-income countries is a potential limitation to our findings. Therefore, our results should be interpreted with caution. Although the data regarding the immunization data quality—in low- and middle-income countries—are limited in the literature, coverage values were found to be inflated and adjusted in the official reports (Harrison et al. 2020). In addition, many other logistic deficiencies were reported including healthcare worker-related factors, the fragmentation of health information system, bad data management, and targets’ overreliance (Harrison et al. 2020). Akin to that are the problems in the costs and cost-effectiveness of the immunization coverage in low- and middle-income countries (Munk et al. 2019).

## Conclusion

In conclusion, our analysis provides novel insight into the potential correlation between the coverage rates of universal poliovirus vaccination policies and the mortality rates/new cases of COVID-19 infection. Countries with increased poliovirus vaccination coverage rates are significantly correlated with increased mortality rates and the number of new cases of COVID-19 per one million population. In the same context, high-income countries had significantly higher mortality rates and an increased number of new cases of COVID-19 per 1 million populations. Finally, countries that implemented poliovirus vaccination policies earlier had significantly more mortality cases and an increased number of new cases of COVID-19 per 1 million inhabitants.

## Supplementary information

ESM 1 (DOCX 15 kb)

## Data Availability

Data are available on request.
