# Supplementary material for: Correlation between polio immunization coverage and overall morbidity and mortality for COVID-19: an epidemiological study
Source: Environ Sci Pollut Res Int. 2021 Mar 2;28(26):34611–8. doi: 10.1007/s11356-021-12861-6 (PMC7923406; doi:10.1007/s11356-021-12861-6)
Supplement: Supplementary file 1 — (DOCX 15 kb) [file 11356_2021_12861_MOESM1_ESM.docx]

**Dwass-Steel-Critchlow-Fligner pairwise comparisons**

| **Supplementary Table 1. Pairwise comparisons - Polio_immunization_coverage** | | | | | | | |
| --- | --- | --- | --- | --- | --- | --- | --- |
|  |  |  |  |  |  |  |  |
| **World Bank Group** | | **World Bank Group** | | **W** | | **p** | |
| High |  | Upper-middle |  | -7.12 |  | < .001 |  |
| High |  | Lower-middle |  | -9.73 |  | < .001 |  |
| High |  | Low-income |  | -10.25 |  | < .001 |  |
| Upper-middle |  | Lower-middle |  | -5.65 |  | < .001 |  |
| Upper-middle |  | Low-income |  | -8.09 |  | < .001 |  |
| Lower-middle |  | Low-income |  | -3.64 |  | 0.050 |  |
|  | | | | | | | |

| **Supplementary Table 2. Pairwise comparisons - Total_Cases_Per_1M_pop** | | | | | | | |
| --- | --- | --- | --- | --- | --- | --- | --- |
|  |  |  |  |  |  |  |  |
| **World Bank Group** | | | | **W** | | **p** | |
| High |  | Upper-middle |  | -9.78 |  | < .001 |  |
| High |  | Lower-middle |  | -11.10 |  | < .001 |  |
| High |  | Low-income |  | -10.51 |  | < .001 |  |
| Upper-middle |  | Lower-middle |  | -7.05 |  | < .001 |  |
| Upper-middle |  | Low-income |  | -8.80 |  | < .001 |  |
| Lower-middle |  | Low-income |  | -3.57 |  | 0.057 |  |
|  | | | | | | | |

| **Supplementary Table 3. Pairwise comparisons - Deaths_Per_1M_pop** | | | | | | | |
| --- | --- | --- | --- | --- | --- | --- | --- |
|  |  |  |  |  |  |  |  |
| **World Bank Group** | | | | **W** | | **p** | |
| High |  | Upper-middle |  | -7.00 |  | < .001 |  |
| High |  | Lower-middle |  | -9.41 |  | < .001 |  |
| High |  | Low-income |  | -8.54 |  | < .001 |  |
| Upper-middle |  | Lower-middle |  | -5.65 |  | < .001 |  |
| Upper-middle |  | Low-income |  | -6.22 |  | < .001 |  |
| Lower-middle |  | Low-income |  | -1.90 |  | 0.534 |  |
|  | | | | | | | |
